# Changes in Weight Loss, Health Behaviors, and Intentions among 400 Participants Who Dropped out from an Insurance-Sponsored, Community-Based Weight Management Program

**DOI:** 10.1155/2016/7562890

**Published:** 2016-06-20

**Authors:** Sam J. Zizzi, Jana Lima Fogaca, Tammy Sheehy, Myia Welsh, Christiaan Abildso

**Affiliations:** ^1^WVU College of Physical Activity and Sport Sciences, Morgantown, WV 26501, USA; ^2^WVU School of Public Health, Morgantown, WV 26501, USA

## Abstract

The majority of weight management research is based on data from randomized controlled studies conducted in clinical settings. As these findings are translated into community-based settings, additional research is needed to understand patterns of lifestyle change and dropout. The purpose of this study was to examine reasons for and consequences associated with dropout (or removal) from an insurance-funded weight management program. Using a mixed methods approach with objectively measured changes in body weight and attendance along with quantitative and qualitative survey data, patterns of intention and behavior change were explored. The results from a sample of 400 respondents support the idea that there are both positive and negative consequences of program participation. Overall, 1 in 5 respondents lost a clinically significant amount of weight during the program (>5% of baseline body weight) and 1 in 3 experienced a positive consequence, while only 6% expressed a negative outcome of participation. Additionally, nearly 90% of all of the consequences that emerged from the data were positive. Attitude change was a major theme, including positive health intentions, perceived success, learning skills, and new appreciation of exercise.

## 1. Introduction

Much of the extant research on weight loss and weight management in adults has focused on the factors associated with adherence during, or after, programs which are typically staged in academic or clinical settings, using randomized controlled designs [1, 2]. Despite the controlled settings and the high level of staff training in these programs, mean dropout from such interventions remains at 20–40% [3, 4] despite incentives to complete regular assessments and frequent follow-up contact from staff members. Therefore, the accuracy of dropout rates reported by such interventions may be misleading, and these studies may not help researchers understand the process of dropout in community-based settings. To evaluate and understand attrition in community-based settings, Grave et al. [5] examined the predictors of dropout from the QUOVADIS observational study of community-based weight loss programs in Italy. Grave and colleagues addressed an important research question: “Are all drop-outs treatment failures?” Findings showed that, despite significantly lower mean percentage weight loss than program completers at 36-month follow-up, dropouts achieved encouraging outcomes. For example, “satisfied” or “confident” dropouts reported a higher mean percentage weight loss of 9.6% and 6.5%, respectively, than continuers (5.2%).

Other studies have shown that program participants who disengage may still join other behaviors or programs. For instance, Ecclestone et al. [6] tracked elderly participants in a community center for three years and noticed that 21% of participants tried out or transferred between programs within the center, while others concurrently participated in exercise programs outside of the center. In addition, Stiggelbout and colleagues [7] found that 31% of older adults who dropped out of a diverse range of organized exercise programs simply switched to another type of exercise. Thus, people who drop out from a program at one stage of their life may return to the same program after more than a year away, or after addressing personal or health problems, or they may express intentions to return in the future [6].

Research examining other health behaviors, such as smoking cessation, has shown that it can take a sequence of repeated, unsuccessful attempts before long-term healthy behaviors are maintained [8]. For example, related research has found that, following short-term commercial weight management interventions (of six months or less), some participants report strong intentions in maintaining newly learned behaviors, increased self-efficacy in achieving a future weight target, and positive changes in habitual physical activity at one-year follow-up [9]. It is possible, therefore, that unsuccessful attempts can be part of the path to a healthy lifestyle because they contain some useful lessons in health education (i.e., exercise technique and eating preferences), stress management, or self-regulation.

As weight management programs increasingly become translated into community settings, a certain degree of dropout can be expected as participants manage multiple time commitments and as program staff members manage multiple job responsibilities. Translation into community settings will bring along with it a greater need for accountability and compliance. Private fitness centers, health departments, and public and private insurers will want to know if these programs are sustainable or profitable. For example, programs established by health insurance agencies tend to have compliance guidelines to which participants must adhere to remain eligible to receive subsidized services. Fitness centers and community clinics benefit from these subsidized programs because they earn money by providing professional services to the policy holder. The main reason for stringent compliance guidelines is that providing such programs can be costly, particularly for insurance-funded programs, which aim to address various health risks that impact chronic disease and curb insurance expenses associated with chronic disease [10]. Thus, a “dropout” from an insurance-sponsored program may either voluntarily quit the program or be administratively removed due to noncompliance. Little is known about the reasons for, and consequences of, dropout from insurance-sponsored health promotion programs. Understanding more about these patterns of behavior may help insurance agencies maximize the benefit of the programs they deliver in communities while keeping costs at a reasonable level. Finally, if positive consequences of dropout are found, these data will help support the continued spending of public and private insurance dollars on health promotion programs.

Thus, the purpose of this study was to examine reasons for and consequences associated with dropout (or removal) from a state-wide, insurance-funded weight management program. The research questions included the following: (1) What are the reasons people drop out from an insurance-funded weight management program? (2) What are the positive and negative consequences associated with dropout? and (3) What are the differences in consequences and future health behavior intentions for those who drop out early, middle, or late into the program?

## 2. Method

### 2.1. Study Setting

West Virginia is a small, Appalachian state ranked among the least healthy states in the US based on adult prevalence of obesity (33% versus 27.5% US Median), diabetes (12% versus 8.7% US Median), and heart disease (6% versus 4% US Median), all of which rank in the top 10 in the United States [11]. The estimated direct medical costs associated with obesity in West Virginia (WV) increased by $51 million USD between 2001 and 2009 [12], which is a meaningful increase for a state with a population of just under two million. These negative health trends, and the economic consequences of them, have driven the need for programs which can help curtail the growth of obesity and promote healthy lifestyles. To combat these trends, the state's largest public insurer, the West Virginia Public Employees Insurance Agency (PEIA), has established a comprehensive weight management program to provide policy holders with opportunities to learn how to be physically active and eat a healthful diet.

### 2.2. The West Virginia PEIA Weight Management Program

The program is a two-year benefit offered by PEIA for people with a BMI of 25 kg/m^2^ or higher or an eligible waist circumference (i.e., 35 inches or greater for women and 40 inches or greater for men). The program, which requires a $20 monthly copay, includes access to a fitness facility in the local community and services provided by a personal trainer, dietitian, and exercise physiologist. Previous single-site and large-scale evaluations of this program's effectiveness have shown low reach, moderate to high effectiveness in short-term and long-term weight loss, and strong potential for sustainability [10, 13–15].

The requirements for ongoing participation (for up to two years) are maintaining at least eight monthly visits to the fitness facility, attending appointments with professionals, and showing improvements in weight or other measured health parameters. Participants are allotted specific minutes of professional services each month in the program. For example, in the first six months, participants receive 120 minutes of personal training per month, three 30–60-minute consultations with a dietician, and two 60-minute fitness assessments by an exercise physiologist. However, there is no standard model of service delivery as each professional is allowed the freedom to deliver fitness and dietary services within their own scope of practice. Facility staff are responsible for entering objective data on participant attendance and body measurements (i.e., waist, weight, body fat, and blood pressure) on a monthly basis into a secure, web-based platform used for evaluation and billing.

Participant compliance with program requirements is reviewed on a bimonthly basis. Those participants that do not meet the eight visits per month minimum attendance criteria for two or more consecutive months are removed from the program. Therefore, participants may be administratively removed for noncompliance with these requirements or other issues may arise that cause the termination of their program (e.g., medical issues and change of insurance). In addition, participants may voluntarily choose to drop out from the program for personal reasons. When they are removed or choose to drop out from the program, they receive a letter informing them of their change in status. One month later, they receive an invitation to complete a program evaluation survey.

### 2.3. Participants and Sampling Method

The study used a mixed methods design including quantitative data on objective outcomes (i.e., length in the program, % weight loss) as well as self-reported reasons and consequences in both quantitative and qualitative forms collected from the program evaluation survey. All program participants over the age of 18 who exited the program during 2014 (*n* = 973) received the program evaluation survey containing questions related to their participation in the program. The survey was sent first via email through a unique link to the participants who provided their email addresses at the start of the program. The electronic version of the survey was sent twice via Survey Monkey with a one-week interval between the first attempt and the reminder. If they did not respond to the email survey, a hard copy of the survey was mailed, which is the first step for participants without an email address. After a week without response from the paper-based survey, a reminder post card was sent to the participant. Using this Dillman [16] modified recruitment method, 400 of 973 participants responded to the survey resulting in a 41% response rate.

The program evaluation survey sent to the participants included questions regarding their reasons for dropping out of the program, their satisfaction with different services of the program, and their future intentions. First, all participants were asked to choose between the reasons for leaving the program from “I chose to withdraw,” “I was removed by program administration,” or “other” and were asked “why have you chosen to leave the weight management program at this time?”

Next, two sets of questions assessed participants' satisfaction with different services of the program. These questions asked participants to rate their exercise facility, personal training, and dietary services using a scaled response from 1 (not at all satisfied) to 4 (completely satisfied). The final question analyzed in this study inquired about the participants' intentions of carrying out the following health-related behaviors in the future, in a categorical format (Yes, No, or I don't know): (a) continue as a private fitness member at the facility; (b) exercise for 30 minutes per day, five days per week; (c) exercise for 60 minutes per day, five days per week; (d) seek out a registered dietician; (e) enroll in a group exercise program; or (f) enroll in a group nutrition program.

After inquiring about their future intentions, two demographic questions (i.e., gender and date of birth) preceded a final question where they could express other thoughts about the program. Because of the wording of this last question, many of its responses were related to satisfaction or dissatisfaction with the program (i.e., “Do you have anything else you would like to add about your level of satisfaction so far with the weight management program and the different services you have received?”). In addition to satisfaction/dissatisfaction, participants also tended to express the positive and negative consequences related to their participation in the program and thus these data were included in the subsequent qualitative analysis.

### 2.4. Data Analysis

First, a frequency analysis of the participants' characteristics was carried out. Then, a frequency analysis of their overall intentions for future health-related behaviors was performed. Finally, the two open-ended questions were analyzed through content analysis [17] to address the research questions. For the analysis of the open-ended data, two researchers independently analyzed and open-coded different subsamples of the data [18] using descriptive coding [19]. Subsequently, they met, contrasted their codes, and agreed on pattern codes (i.e., categories) that provided more meaningful and parsimonious units of analysis [18]. These categories and their codes were organized into a coding book that served as a source for the later provisional coding of the entire sample, allowing a combination of inductive and deductive coding [19]. The software program NVivo 10 was utilized for this step, which allowed for a check of the interrater reliability. Subsequently, coding disagreements were discussed to reach a consensus. A near identical process was used to analyze the consequences and dropout open-ended items. However, data from the items were analyzed independently, that is, by separate sets of researchers and using separate coding books. Among all codes across both analyses, the average interrater reliability between coders was 99%. The index of agreement (kappa) was .65 for the consequences data and .78 for the reasons data. These values are considered moderate to strong agreement values. In any instance where the two raters disagreed, a third rater was engaged so that consensus could be reached in each coding moment before finalizing the code.

For the final research question, respondents were classified into three groups depending on the timing of their program exit: (1) “early” drops (participation of 6 months or less); (2) “mid” drops (between 7 and 12 months of participation); and (3) “late” drops (between 13 and 24 months of participation). Two-way chi-square analysis was used to explore the future intention data as well as the patterns of consequences experienced across the three groups. These bivariate analyses were used to determine if there was any relationship between the length of time in the program prior to exit and their coded consequences and stated intentions. Effect size estimates for all analyses are reported as the contingency coefficient, and values >.3 can be considered moderate.

## 3. Results

The respondents were aged between 24 and 70 years, with a mean age of 48.6 years (SD = 11.1). The mean length of participation in the program was 9.8 months (SD = 5.6), with 33.7% exiting the program within the first six months, 35.7% exiting between 7 and 12 months, and 30.6% completing 13–24 months. Among the male participants, mean weight at the beginning of the program was 262.2 lbs (SD = 57.3), while women had a mean weight of 210 lbs (SD = 49.6). Men's mean waist circumference at the beginning of the program was 46.9 inches (SD = 6.9) and women's was 42.4 inches (SD = 9.8). On a 6-point Likert scale, with one meaning “not at all satisfied” and six meaning “completely satisfied,” participants had a mean satisfaction of 4.4 (SD = 1.5). At the time of survey completion, the average percent body weight loss for the respondents was 2.27% (SD = 4.9). Additionally, upon program exit, 21% of respondents lost at least 5% of their baseline body weight during the course of the program, while 26.7% had gained weight.

To check for demographic differences between the survey respondents and nonrespondents, a series of independent samples *t*-tests was conducted to compare the groups on characteristics at baseline. These analyses were conducted separately for men and women. Analyses indicated that mean BMI, waist circumference, body fat percentage, and length of participation in the program of participants who dropped out from the program and responded the survey were not significantly different from the participants who dropped out and did not respond the survey (*p* > .05). However, the nonrespondents' age (M = 44.6, SD = 11.6) was significantly lower than the respondents' (M = 48.6, SD = 11.1), *t*(971) = 5.33, *p* < .001. Despite this small difference in age, survey respondents and nonrespondents appear to report similar profiles related to their weight and program participation, thus reducing the concern for selection bias in the subsample of respondents.

### 3.1. Self-Reported Reasons for Dropout

**Figure 1:**
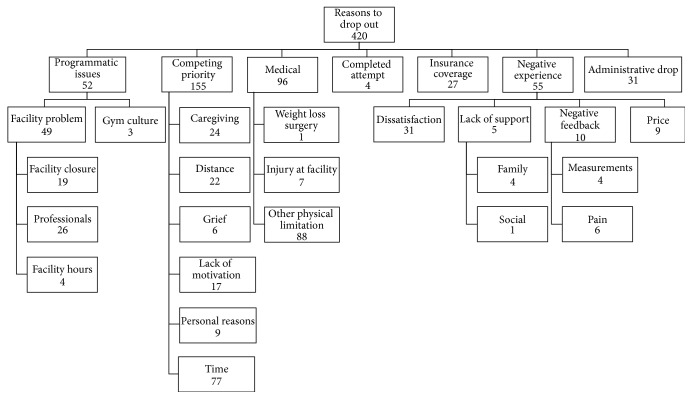
Themes for reasons to drop out. This figure illustrates the themes that emerged from the analysis of the answers of 375 participants to the question about their reasons to drop out from the program and the frequency that they were coded.

Among the 400 survey respondents, 375 participants responded to the question about their reasons to drop out from the program, while 272 responded to the final open-ended question. Reasons for dropout were allowed to emerge from either of these two questions. In total, reasons to drop out from the program were coded 420 times, with some participants indicating more than one reason and some leaving the question blank. Seven major themes regarding the participants' reasons to drop out from the program emerged from the survey responses: competing priority (36.9%), medical (22.8%), negative experience (13.1%), programmatic issues (12.4%), administrative drop (7.4%), insurance coverage change (6.4%), and completed attempt (1.0%).  Figure 1 shows these themes and their subthemes.

The competing priority theme was the largest emergent theme with 155 occurrences (36.9%) and included the subthemes of time (18.3%), caregiving (5.7%), distance (5.2%), lack of motivation (4.1%), personal reasons (2.1%), and grief (1.4%). Time was a frequently coded subtheme, including statements of limited or lack of time to comply with the program requirements (e.g., “not enough time at the moment”). Caregiving meant having to take care of older family members (e.g., “family commitments due to sick family member”) or children (e.g., “my child playing sports kept me from coming at the scheduled times of the classes that I enjoyed taking”). Distance included the gym being far from home or work and moving to another place. Lack of motivation included need for external motivation, not being able to meet requirements, and just not wanting to participate anymore (e.g., “I was no longer invested in the program and wanted to do something else”). Personal reasons contained personal or family reasons that were not specified (e.g., “personal family difficulties were drawing me away from working out regularly”). Finally, grief regarded difficulties in adherence due to grieving the death of a close person (e.g., “death of my spouse, so I haven't adhered as well as I should have”).

The medical reasons theme included other Physical Limitation (20.9%), injury at facility (1.7%), and weight loss surgery (0.2%). Other physical limitation was the most frequently coded subtheme and included a variety of health issues other than injury at facility and weight loss surgery (e.g., “I have been diagnosed with blood clots in the lungs and cannot work out for several months until I heal”). Getting injured at the facility was reported by seven respondents, which prevented further exercise participation (e.g., “I injured myself during a class”). Weight Loss Surgery (e.g., “bariatric surgery”) was indicated by one participant.

The theme negative experience (13.1%) included the subthemes of dissatisfaction (7.4%), negative feedback (2.4%), price (2.1%), and lack of support (1.2%). Dissatisfaction included disappointment with results, training, and services in general (e.g., “I felt like the program wasn't meeting my needs”). Negative feedback included negative feedback from measurements (1.0%) and pain (1.4%). The first could be exemplified as “the negative feedback made me feel like a failure instead of acknowledging my improvement, so I quit going,” while the second involved any kind of pain related to the participation of the program (e.g., “I was experiencing severe leg pain”). Price comprised unwillingness or impossibility of paying for direct or indirect costs of the program (e.g., “cost of daycare while I participate in the program”). Finally, lack of support included family and social support. Lack of family support (1.0%) was coded when family members were unsupportive of the participant's compliance with the program (e.g., “conflict with my spouse, he felt I was spending too much time at the gym”), while lack of social support (0.2%) contained only one respondent who wished to have a buddy exerciser: “I needed to have someone to work out with!”.

Programmatic issues was divided into the subthemes facility problem (11.7%) and gym culture (0.7%). The subtheme facility problem was further divided into professionals (6.2%), facility closure (4.5%), and facility hours (1.0%). The professionals subtheme regarded problems with the staff delivering the services offered by the program, such as trainer's or dietitian's ineffectiveness, limited availability, and lack of support (e.g., “didn't feel I had the support needed from staff”). Facility closure represented when the gym closed or stopped offering the program and this situation triggered the participants' dropout (e.g., “the facility closed and I chose not to select an alternate facility”). Finally, facility hours included dissatisfaction with the facility hours (e.g., “hours at the facility did not coincide with my work hours”). Gym culture included expressions of uncomfortable feelings due to the gym environment (e.g., “[I] did not like the gym environment”).

The themes completed attempt, insurance coverage, and administrative drop (14.8% total response among the three categories) were less meaningful since they involved administrative issues. Insurance coverage contained participants who left for another job or retired, thus losing the insurance benefits, and administrative drop encompassed respondents who were removed by the program administration due to noncompliance. Completed attempt included people who believed that they had already completed the program and ceased going to the facility.

### 3.2. Self-Reported Consequences of Program Participation

**Figure 2:**
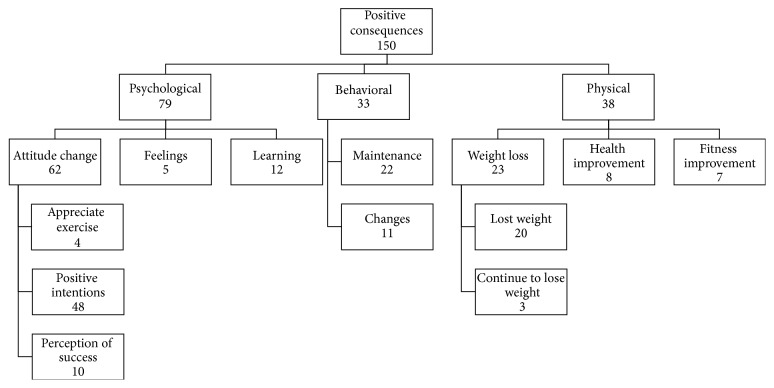
Positive consequences theme and its subthemes. This figure illustrates the subthemes emerged within the positive consequences theme and the frequency in which they were coded.

**Figure 3:**
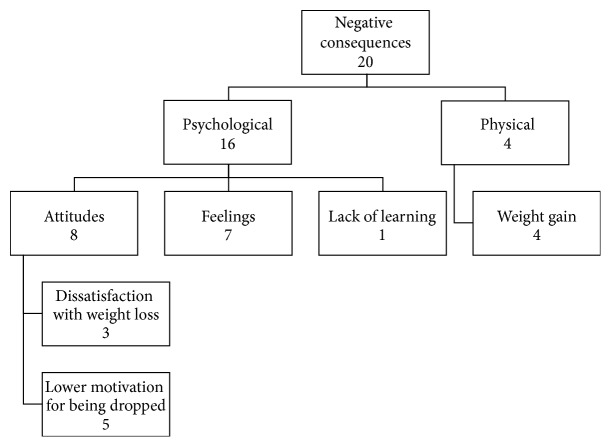
Negative consequences theme and its subthemes. This figure illustrates the subthemes emerged within the negative consequences theme and the frequency in which they were coded.

Among the 400 survey respondents, 272 responded to the final open-ended question about their experiences in the program. Two major themes emerged from these 272 responses: positive and negative consequences from participation in the program. Positive consequences were coded 150 times (88.2%) and negative consequences 20 times (11.8%). Positive consequences included three subthemes: psychological (52.7%), behavioral (22.0%), and physical (25.3%). These subthemes were further divided into smaller categories, as shown on Figure 2. The negative consequences were composed of two subthemes: psychological (80%) and physical (20%) consequences. The negative psychological consequences also had subthemes, as seen in Figure 3.

Three themes emerged in the positive psychological consequences. The first was attitude change (41.3%), which included the codes positive intentions (32%), perception of success (6.7%), and appreciate exercise (2.7%). The most common subtheme within the attitude change was positive intentions, which comprised intentions such as continuing to exercise, joining another weight management or exercise program, and seeking nutrition guidance (e.g., “I will continue to work at home on my own equipment”). The second most common was perception of success, including subjective comments regarding achieving success (e.g., “I have worked very hard and have had great success”). The last subtheme within the attitude change was appreciate exercise, which included liking exercise and realizing positives of exercising (e.g., “the program made me realize how good I feel when I do work out”).

Another subtheme included in the positive psychological consequences was learning (8%), which contained several learning experiences, such as learning about exercise, eating, self-motivation, and weight loss (e.g., “I learned a great deal from both the trainer and dietitian I have continued to utilize info from both”). Finally, the last subtheme within the positive psychological consequences theme was feelings (3.3%), which contained general positive feelings such as being proud (e.g., “I'm very proud of myself for what I have accomplished with some help from the program”).

The second major theme within the positive consequences was behavioral (22%), which comprised maintenance (14.7%) and changes (7.3%). Behavioral maintenance included continuing to exercise and eating healthy (e.g., “As of right now I am once again working on my fitness and diet on my own”). Behavioral changes referred to changes in exercise and eating habits without referring to the maintenance of these changes (e.g., “I have changed my eating and exercise habits since I began [the] program”).

The final major subtheme within the positive consequences was physical (25.3%), which included the subthemes weight loss (15.3%), health improvement (5.3%), and fitness improvement (4.7%). Weight loss subtheme was subcoded into lost weight (13.3%) and continue to lose weight (2%), which differed regarding when the weight loss happened (during the program or after dropping out of it). An example of lost weight is “I lost 45 pounds in the program” and one of continue to lose weight is “since leaving the program I have lost about 15 pounds.” Health improvement regarded improvements in health-related markers (e.g., hemoglobin A1C), stress, energy, and pain (e.g., “while working through this program my A1C level dropped from 7 to 5.7”). Fitness improvement was related to enhancement in physical capability such as strength, flexibility, stamina, and resistance (e.g., “6 months ago it took me 30 minutes for a mile. Now I can do 3 miles in 31 minutes”).

Among the negative consequences (*n* = 20), the psychological consequences was the most frequent coded subtheme and was further subcategorized into attitudes (40%), feelings (35%), and lack of learning (5%). Attitudes included dissatisfaction with weight loss (15%) and lower motivation for being dropped (25%). The first comprised expressions that weight loss was not enough to satisfy the participant (e.g., “I only lost 10–15 lbs and after I felt that wasn't enough”). The latter regarded the participant's decrease in motivation as a consequence of being removed from the program (e.g., “I was kicked out of the program twice, it has been very discouraging”). Still within the negative psychological consequences, the code feelings included negative feelings such as guilt, disappointment, and shame (e.g., “I feel guilty for not continuing exercising as faithfully on my on”). Finally, the last negative psychological consequence was lack of learning and it was coded once: “I didn't really learn anything new or take away anything useful.” The physical negative consequences were comprised of only one subtheme: weight gain (20%). It included statements of weight gain during the program or after leaving it (e.g., “I actually gained more weight after enrolling this program”).

### 3.3. Comparing Future Intentions and Consequences across Groups

Regarding the respondents' (*n* = 400) intentions for future health-related behaviors, 64% intended to exercise 30 minutes per day, five days per week, 16% intended to exercise 60 minutes per day, five days per week, 11% intended to join a group exercise program, and 21% intended to continue as a private member of the fitness facility that they were attending during the program. Additionally, 7% intended to seek the help of a registered dietitian to make nutrition changes and 13% intended to enroll in a group nutrition program. When comparing these intentions among respondents who dropped out of the program early (6 months or less of participation), in the middle (between 7 and 12 months), or later (between months 13 and 24), two trends in the data were found. Chi-square analysis showed that respondents who stayed for seven or more months in the program were more likely to intend to continue exercising five days a week for at least 30 minutes (68.6% combined for the mid and later groups) compared to early dropouts (55%); however this effect was not significant, *χ* ^2^(4, *N* = 382) = 7.09, *p* = .131, cc = 0.14. This trend was not found for intentions to exercise five days a week for 60 minutes, which overall remained low in the overall sample (16.1%). However, early dropouts (8.2%) were significantly more likely to intend to seek a dietitian help to make weight changes than middle dropouts (5.8%), *χ* ^2^(4, *N* = 371) = 12.97, *p* = .011, cc = 0.18. The pattern of intentions to seek out group exercise classes or group nutrition services did not differ in a meaningful way across the three groups. In general, exercise intentions were substantially higher than dietary intentions.

The final set of chi-square analyses targeted the relationship between stage of dropout (early, middle, and late) and consequences expressed. First, two analyses were conducted to see if the percentage of respondents reporting positive or negative consequences differed across the three groups. Subsequent analyses explored the various subthemes of positive and negative consequences across the groups. Positive consequences experienced in the program did show an increasing trend across the three groups, from 30.4% to 31.3% to 38.8%, but this relationship was not significant *χ* ^2^(2, *N* = 268) = 1.59, *p* = .451, cc = 0.07. Overall, one-third of those who answered this open-ended question reported a positive consequence of program participation.

The largest differences in reported positives themes were from the psychological category. Those participants who dropped out after 12 months (32.5%) were nearly twice as likely to report a positive psychological outcome compared to those who dropped out early (17.4%); however, this small effect failed to reach significance, *χ* ^2^(2, *N* = 268) = 5.30, *p* = .071, cc = 0.14.

A different trend was revealed for the negative consequences, with those who dropped out in the middle group reporting a negative outcome 3–5 times as often (12.5%) as the early (2.2%) or late (3.8%) dropouts, *χ* ^2^(2, *N* = 268) = 9.723, *p* = .008, cc = 0.19. This pattern held true for all of the subthemes of negative consequences, with the exception of “dissatisfaction with weight loss.”

## 4. Discussion

The results of this mixed methods study support the idea that there are both positive and negative consequences experienced in a sample of weight management program dropouts. Overall, 1 in 5 respondents lost a clinically significant amount of weight during the program (>5% of baseline body weight), regardless of the time of program exit, and 1 in 3 experienced a positive consequence, while only 6% expressed a negative outcome of participation. Additionally, nearly 90% of all of the consequences that emerged from the data were positive. Thus, some of the dropouts appear to have experienced substantial success in terms of outcomes or lessons learned through participation, and five times as many respondents reported a positive consequence compared to those who reported a negative consequence of participation. In particular, the psychological consequences seem meaningful. Attitude change and skill acquisition were major themes, including positive health intentions, perceived success, learning skills, and new appreciation of exercise. These findings support previous theories of health behavior change including the theory of planned behavior's connection between intentions, self-efficacy, and past behavior [20] as well as similar work on barriers to the self-management of diabetes [21]. Specifically, based on these models, if programs can help individuals increase their health intentions, self-efficacy, and specific behavioral skills, regardless if they dropout or not, then they will increase the probability of future health behavior change among participants.

### 4.1. Future Health Intentions

Approximately two-thirds of the respondents indicated they intend to continue exercising five days a week for 30 minutes, an amount of physical activity that would meet guidelines for general health [22]. Some respondents may have received as much out of the program as they needed, and they felt ready to move onto a self-managed program. Others simply learned some new skills and experienced a change in attitude toward eating or physical activity. Since intentions are widely regarded as being the closest psychological predictor of future behavior, it is possible that if these intentions are sustained, a subsequent attempt at behavior change will stick [6, 23]. Thus, the “failure” of dropout may have resulted in a valuable learning experience where participants identified activities they enjoyed and learned new meals to prepare for themselves and their families. These findings support previous research that also found positive intentions among former program participants [9, 24] and research that has identified a subset of dropouts who may have experienced substantial success [5]. Additionally, it is possible that a shift in identity occurred moving some former participants towards a newer, active identity [25]. Using self-determination theory as another frame of reference, if participants of health behavior interventions can develop more intrinsic forms of motivation, by identifying activities they enjoy and that confirm their sense of self, their future efforts may be sustained over a longer period of time.

### 4.2. Comparing Positive and Negative Consequences of Participation

Not all participants reported positive outcomes, however, and approximately 1 in 4 of the dropouts gained weight during the program. It is important to note that most of the negative consequences reported were also psychological in nature (including lower motivation and dissatisfaction with weight loss), and there were no strong themes for things such as “injury” or “worsened disability.” Integrating the objective and subjective data on consequences allows an observation that approximately 20% (percent of sample with clinically significant weight loss) to 33% (percent of respondents who self-reported a positive consequence) of dropouts of this community-based weight management program experienced a meaningful positive consequence resulting from program participation. Future research may consider capturing some of these possible benefits along with reporting the outcomes of those who complete intervention programs.

### 4.3. Adding Behavioral Services during High Risk Periods for Dropout

The average respondent dropped out approximately 10 months into this two-year-long program, and negative consequences were reported at a higher rate among middle dropouts compared to early or late dropouts. This particular phase of the program (between 7 and 12 months of participation) appears to be the highest risk of dropout and may warrant additional attention. Competing priorities (i.e., lack of time) was the most widely cited reason for program exit (noted by 37% of respondents), suggesting that the working adults in the program often struggle to manage the multiple commitments of work, family, and health. This evidence supports the idea that some people need more support than others based of the timing of their attempt and the strength of their motivation at program entry. These data also support the finding from Grave et al. [5] that some participants may need help managing “logistics” to increase long-term adherence.

In this particular program, there is also a significant change to the services provided to participants during this time frame. In particular, their access to personal training drops from 120 minutes per month to 60 minutes per month, and they are not scheduled for another fitness or dietary reassessment until month 13. This reduction in professional services and the lack of accountability that comes along with those reductions may cause some participants to lose motivation and commitment. It is also possible that adhering to the facility-based program that requires at least eight in-person visits per month is not sustainable for everyone over two years. Anecdotally, many participants report traveling substantial distances to reach the facility, particularly in rural parts of the state. Adding further support in the form of behavioral services may be needed considering that many program enrollees enter with various comorbidities or physical limitations. This issue highlights a key difference in populations who are enrolled in community-based programs compared to clinical trials; prior to group assignment, many participants with comorbidities are often excluded from trials. Adding behavioral services (in various forms and at various stages of the program) could help participants learn new skills and stay engaged long enough to maintain the multiple health behaviors they have initiated [26].

### 4.4. A Possible Mismatch in Training of Staff and Needs of Participants

The final discussion point worth noting relates to the differences observed when working with high risk adults and translating programs into communities. Though this program is a fitness facility based program, the weight management program participants are often dissimilar from typical gym members. They are typically obese, possess low fitness at baseline, and present with additional health risk factors including hypertension, hyperlipidemia, and/or diabetes. As insurance companies try to build connections with the fitness industry, it is imperative that fitness staff are trained to safely and accurately assess, prescribe modified exercise to, and train high risk clients. A mismatch between the needs of these participants and the staff training may be contributing to weak outcomes, injuries, and program noncompliance. These issues were noted by some participants who dropped out, thus highlighting the difficulty of ensuring high “treatment fidelity” in a large, community-based program.

The results of this study should be interpreted given the following limitations. Despite the analyses indicating the responders are not that different from the nonresponders, there is still the potential for self-selection bias in the data. With a 40% response rate, it is possible that survey responders were more likely to report positive consequences than those who chose not to respond. Next, in our analyses of those who were dropped from the program, we did not differentiate between those who left the program voluntarily and those who were dropped due to noncompliance. However, this difference between groups is not as large as it seems. All participants self-enroll in the program and decide on their own to attend the facility or to stop coming. Thus, both groups are “voluntary” drops. Some participants are conscientious enough to call program staff, and the others simply stop going to the facility and then are dropped within 2-3 months for noncompliance. Finally, the study is limited because there is no follow-up data indicating if the positive intentions expressed at program exit led to future observable behaviors. Future studies will explore these patterns over time and should address the relative importance of the psychosocial and environmental factors and barriers that emerged among participants [27].

There is strength in the study's design and data given the unique mix of quantitative, objectively observed changes in body weight and attendance along with the qualitative reasons, and consequences expressed by several hundred respondents. The loss of internal validity when programs are disseminated into multiple community settings is unavoidable. However, we cannot expect to treat or reverse the obesity epidemic in the US with the limited reach of randomized controlled trials in clinical settings. We must embrace the messiness of community-based programs, especially those funded by insurance agencies, because they are sustainable and possess strong external validity. Findings of the current study can be used to inform and improve future community-based research and multisite intervention programs with similar rural populations in the United States that experience the greatest health disparities in chronic disease.
